# MicroRNA dysregulation in myelodysplastic syndromes: implications for diagnosis, prognosis, and therapeutic response

**DOI:** 10.3389/fonc.2024.1410656

**Published:** 2024-08-02

**Authors:** Ilina Dimitrova Micheva, Svilena Angelova Atanasova

**Affiliations:** ^1^ Hematology Department, University Hospital St. Marina, Varna, Bulgaria; ^2^ Faculty of Medicine, Medical University of Varna, Varna, Bulgaria

**Keywords:** myelodysplastic syndromes, microRNA, diagnostic and prognostic biomarkers, therapeutic microRNAs, circulating microRNA

## Abstract

Myelodysplastic syndromes (MDS) are a group of malignant clonal hematological disorders with heterogeneous clinical course and risk of transformation to acute myeloid leukemia. Genetic and epigenetic dysregulation, including alterations in microRNA (miRNA) expression, plays a pivotal role in MDS pathogenesis influencing disease development and progression. MiRNAs, known for their regulatory roles in gene expression, have emerged as promising biomarkers in various malignant diseases. This review aims to explore the diagnostic and prognostic roles of miRNAs in MDS. We discuss research efforts aimed at understanding the clinical utility of miRNAs in MDS management. MiRNA dysregulation is linked to specific chromosomal abnormalities in MDS, providing insights into the molecular landscape of the disease. Circulating miRNAs in plasma offer a less invasive avenue for diagnostic and prognostic assessment, with distinct miRNA profiles identified in MDS patients. Additionally, we discuss investigations concerning the role of miRNAs as markers for treatment response to hypomethylating and immunomodulating agents, which could lead to improved treatment decision-making and monitoring. Despite significant progress, further research in larger patient cohorts is needed to fully elucidate the role of miRNAs in MDS pathogenesis and refine personalized approaches to patient care.

## Introduction

Myelodysplastic syndromes are a heterogenic group of clonal disorders of the hematopoietic stem cell, characterized by ineffective hematopoiesis, morphological manifestations of dysplasia and high risk of transformation into acute leukemia (1). Numerous genetic and epigenetic factors are involved in the development, course and progression of the disease.

Epigenetics is all the information that is passed on during cell division but is not encoded in the DNA sequence. It refers to the modification of DNA and histone proteins to alter chromatin configuration and gene expression, influencing the regeneration, differentiation and development of hematopoietic progenitor cells. Epigenetic mechanisms include DNA methylation, post-translational modification of histone proteins and chromatin remodeling and synthesis of non-coding RNAs. Disturbances of epigenetic modifications have a key role in the maintenance of regenerative hematopoietic stem cells and leukemic stem cells (2).

MicroRNAs are short non-coding RNAs and changes in their expression levels can contribute to hematological tumor development. MiRNAs have a key role in the regulation of hematopoiesis (3), and their differential expression in MDS is associated with the clinical course of the disease (4–6), and its transformation in AML (7). However, the exact role of miRNAs in the pathogenesis of MDS is still unclear. They participate in the process of oncogenesis through multiple processes leading to the avoidance of angiogenesis, cell differentiation, apoptosis and proliferation of tumor cells (8). In addition, microRNAs also exert epigenetic control by influencing other epigenetic mechanisms - such as DNA methyltransferases, leading to global hypomethylation, or by overexpression of some enzymes of the PcG complex such as EZH2, and thus can function as oncogenes and tumor suppressor genes. Significant levels of microRNAs have been found in a number of body fluids such as plasma, serum, urine, breast milk and saliva. Specific panels of microRNAs have been identified as being associated with the diagnosis and outcome of a number of diseases and especially malignant ones. The role of microRNAs as diagnostic, prognostic and predictive markers in myelodysplastic syndromes is a subject in our analysis.

## Biosynthesis of microRNAs

MicroRNAs are non-coding endogenous RNAs with a length of 19-25 nucleotides that are complementary to the 3’ untranslated region of the target genes. The main enzyme responsible for their transcription is RNA polymerase II, but this process is influenced by numerous other genetic and epigenetic factors such as the tumor suppressor gene p53 and CpG methylation (8). RNA polymerase II leads to the synthesis of a primary miRNA (pri-miRNA), which has a 5’ cap and a 3’ poly(A) tail. It is further processed by a microprocessor complex composed of ribonuclease III, Drosha and DGCR8 (DiGeorge syndrome critical region 8), forming precursor miRNA (pre-miRNA), which is exported from the nucleus to the cell cytoplasm by exportin 5. There it is recognized by Dicer, which cuts the pre-miRNA to a fixed length, usually 21-25 nucleotides. The final product is a mature miRNA that is bound to a specific protein Argonaute (AGO) and thus an RNA induced silencing complex (RISC) is formed. Four AGO proteins are found in humans. The miRNA in the RISC complex binds to messenger RNA and this leads to translational repression. In addition, RISC also acts directly on ribosomal subunits by preventing the 60S from joining the translation complex or by inhibiting the formation of the 80S complex. There is another mechanism for mRNA repression by miRNAs through deadenylation of mRNAs by RISC. A small fraction of miRNAs can also be synthesized independently of Drosha and Dicer. A Drosha-independent pathway is enabled by the synthesis of mirtrons (9). Mirtrons are encoded in an intronic region and thus precursors are generated by mRNA splicing mechanism that does not require Drosha. The mirtrons are then post-processed by Dicer. A Dicer-independent pathway in the synthesis of miRNA-451 (10, 11), has also been described. After Drosha-dependent processing, a structure containing 18 nucleotides is generated, which is too short for Dicer-dependent processing. Instead, pre-miRNA-451 directly binds to RISC, where AGO2-dependent cleavage generates ac-pre-miR-451 (AGO-cleaved pre-miR-451). This structure is further processed by a specific ribonuclease and thus mature miRNA-451 is synthesized (12).

## MicroRNAs in myelodysplastic syndrome as diagnostic and prognostic biomarkers

The field of microRNAs in MDS is rapidly evolving, revealing their dysregulation as a hallmark feature of this disease. These small non-coding RNAs exhibit differential expression patterns in MDS, reflecting the underlying molecular mechanisms driving disease pathogenesis. As such, miRNAs hold immense potential and offer insights into disease subtype classification and patient outcome prediction. There is an increasing number of studies focusing on different microRNAs, highlighting a growing interest in their potential as diagnostic and prognostic biomarkers in MDS (**Table 1**).

**Table 1:** Diagnostic and prognostic microRNAs.

MicroRNA	Sample source	Expression	Function	Target	Implication	Reference
MiR-16	Bone marrow	Upregulated	Tumor suppressor	VEGF	Prognosis	(13)
MiR-22	Bone marrow/Plasma	Upregulated	Role in HSC renewal	TET2	Prognosis	(14)
MiR-146b	Bone marrow	Upregulated	Role in tumorigenesis, erythropoiesis, megakaryopoiesis	TRAF6, IRAK1, PDGFRA	Diagnostic and prognostic marker	(15, 16)
MiR-181	Bone marrow	Upregulated	Regulator of granulocytic and macrophage differentiation	PRKCD, CTDSPL, CAMKK1	Prognostic marker	(7, 17, 18)
MiR-320	Bone marrow	Upregulated			Diagnostic and prognostic marker	(4, 19)
MiR-424	Bone marrow	Downregulated	Regulator of monocyte and macrophage differentiation	VEGFR2	Diagnostic marker, therapy response marker	(15, 20, 21)
MiRs in DLK1-DIO3 genomic region	Bone marrow	Upregulated	Apoptosis, regulation of HSPC differentiation	MEG3-DMR	Prognostic markers, Therapy response markers	(22)
MiR-194	Bone marrow	Upregulated	Apoptosis	p53	Prognostic marker	(4)
MiR-218		Downregulated	Role in tumorigenesis	SLIT2/3	Prognostic marker, Potential therapeutic target	(23)

### MicroRNA-16

A recent study revealed lower levels of miR-16 in bone marrow CD34+ cells obtained from patients diagnosed with high-risk MDS, coinciding with upregulated levels of vascular endothelial growth factor (VEGF) (13). VEGF, a critical angiogenic factor, plays a pivotal role in hematopoietic stem cell regulation (24), and its dysregulation is associated with tumor proliferation and angiogenesis, affecting both endothelial and leukemic cells (25). Consistent findings across several studies have demonstrated the role of VEGF in the pathogenesis of MDS and high expression of VEGF was correlated with increased transfusion needs, overall survival, and leukemia-free survival (26–28). These observations support the hypothesis that apoptosis dysregulation characterizes MDS pathogenesis, with low-risk disease showing heightened apoptosis, while disease progression is marked by acquired apoptosis resistance and aberrant VEGF expression. Xiong et al. further corroborated these findings by verifying miR-16’s direct binding to the VEGF 3’-UTR, suggesting a tumor-suppressive role for miR-16 in MDS development by targeting VEGF (13).

### MicroRNA-22

**Figure 1:**
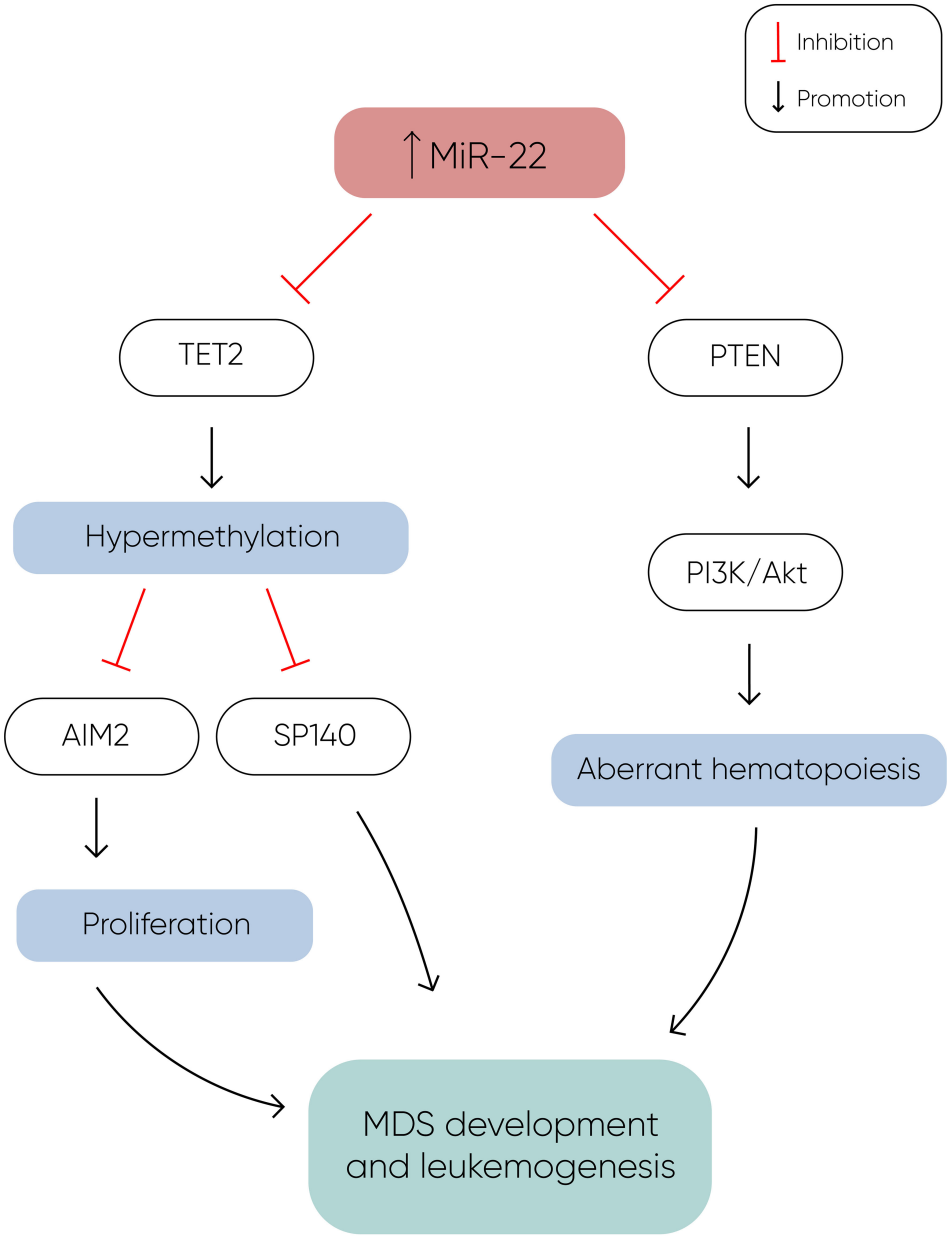
MiR-22 directly inhibits TET2 expression, resulting in hypermethylation and decreased expression of TET2 target genes such as AIM2 and SP140. AIM2 has a role in the reduction of cell proliferation by inducing cell cycle arrest, and along with SP140, plays a crucial role in MDS development and leukemogenesis. Additionally, miR-22 downregulates PTEN expression, a phosphatase that dephosphorylates phosphatidylinositol-3,4,5-trisphosphate (PIP3), thereby counteracting the activation of the PI3K/AKT pathway, which leads to aberrant hematopoiesis. The concomitant silencing of TET2 and PTEN by miR-22 enhances cell proliferation and survival, ultimately contributing to the development of MDS and leukemogenesis (14).

MiR-22 is another miR upregulated in MDS and plays a role in hematopoiesis and hematopoietic stem cell renewal by negatively regulating TET2 protein levels (**Figure 1**) (14). TET2, belonging to the TET methylcytosine dioxygenase family, is crucial for the conversion of 5-methylcytosine (5-mC) to 5-hydroxymethylcytosine (5-hmC) (29), and mutations in TET2 are frequently observed in MDS. Notably, Song et al. demonstrated a direct correlation between elevated miR-22 levels and poor survival rates in MDS patients, independent of factors such as blast count and cytogenetic karyotype. They proved that, by repressing TET2 expression, miR-22 can remodel the epigenetic landscape, resulting in global changes in 5-hmC levels and alterations in the expression of TET2 target genes like AIM2 and SP140. Additionally, miR-22 emerges as a potential therapeutic target in MDS, as inhibition of miR-22 in cell cultures lead to a reduction in their colony forming capability (14). Further studies have highlighted the concordance between miR-22 expression levels in bone marrow CD34+ cells and plasma of MDS patients, with higher expression observed in high-risk disease (30). Interestingly, expression levels of miR-22 tend to increase with disease progression from lower to higher risk stages but decline upon transformation to overt AML, although this trend lacks statistical significance due to limited cohorts. While miR-22 holds promise as a potential biomarker for response to hypomethylating agents due to its targeting of TET2, studies have not found a correlation between treatment response and miR-22 levels (31), suggesting the need for further investigation into its therapeutic implications in MDS.

### MicroRNA-150

MicroRNA-150 is another microRNA with a role in hematopoiesis, which is moderately expressed in megakaryocyte/erythrocyte precursors (32). It is upregulated during megakaryocytic differentiation (32) and downregulated in normal erythropoiesis (33). In a study conducted by Hussein et al., a significant elevation of miR-150 levels was observed in MDS with del(5q) compared to normal hematopoiesis (34). The researchers suggested that miR-150 negatively regulates the expression of the myeloblastosis virus oncogene (MYB), a DNA-binding transcription factor crucial for hematopoietic development. Building on this, a more recent study by Liu et al. confirmed MYB as a direct target of miR-150 in MDS cells and also showed that miR-150 is targeted by the oncogene BC200, also known as brain cytoplasmic RNA 1 (BCYRN1) (35). Their findings demonstrated that the overexpression of BC200 in MDS cells acted as a sponge for miR-150-5p, leading to an increase in MYB mRNA levels, highlighting the intricate regulatory network involving miR-150, MYB, and BC200 in the context of hematopoietic disorders.

### MicroRNA-181

The miR-181 family serves as a crucial negative regulator of granulocytic and macrophage differentiation by directly targeting PRKCD, CTDSPL, and CAMKK1 (36). Dysregulation of the miR-181 family expression is implicated in the pathogenesis of AML, with aberrantly elevated levels believed to contribute to disease development (36). Pons et al. identified miR-181a with progressively increasing expression levels from controls to early-stage MDS, to advanced MDS, and post-MDS AML, suggesting its potential as a prognostic marker for disease progression (7). Moreover, miR-181a-5p, miR-181b-5p, and miR-181d-5p were found to be overexpressed in MDS patients who later progressed to AML, independently of blast count (17). Another study revealed high expression of four miR-181 family members in high-risk MDS, and also that elevated miR-181 expression in low-risk MDS correlated with reduced overall survival (18). Additionally, miR-181c upregulation was observed in GATA2 deficiency (37), a disorder associated with progressive cytopenias, bone marrow hypocellularity, severe immunodeficiency, sensorineural hearing loss, lymphedema, abnormal myeloid differentiation, and increased propensity to develop bone marrow failure, MDS, AML, or chronic myelomonocytic leukemia (38). Contrary to these findings, Liang et al. reported downregulation of miR-181a-2-3p in MDS patients compared to healthy controls, still secondary AML patients exhibited higher expression levels. Interestingly, low levels of miR-181a were associated with worse overall survival in MDS patients, but the analysis excluded patients with secondary AML (39). Nonetheless, these findings present discrepancies that warrant further investigation, possibly attributable to the limitations of small sample cohorts, underscoring the need for larger-scale studies to elucidate the precise role of miR-181 family members in MDS and AML pathogenesis.

### MicroRNA-765

MiR-765 has emerged as another player in the pathogenesis of MDS. A recent study by Kang et al. unveiled elevated levels of this miRNA in MDS patients, particularly those with multilineage dysplasia (40). Functionally, miR-765 exerts its effects by inducing apoptosis. This effect occurs through the downregulation of proteolipid protein 2 (PLP2) (41), an integral membrane protein located in the endoplasmic reticulum. Consequently, the upregulation of miR-765 in MDS leads to apoptosis induction via the inhibition of PLP2.

### MicroRNA-320

Upregulation of the miR-320 family has been observed in MDS patients compared to controls (4, 19). Moreover, elevated levels of miR-320c and miR-320d have been associated with shorter OS in MDS patients and miR-320d has been identified as an independent prognostic factor for OS (19). These findings underscore the clinical relevance of the miR-320 family in MDS management, warranting further investigation into its diagnostic and prognostic value.

### MicroRNA-34a

MiR-34a plays a pivotal role in the pathogenesis of MDS through various mechanisms. Its function as a regulator of proliferation, as miR-34a inhibits cell proliferation by inducing apoptosis, thereby affecting hematopoietic stem cells (42). Additionally, the upregulation of miR-34a, along with miR-155, has been linked to the inhibition of neutrophil migration by downregulating genes such as DOCK8, FGD4, and Rac1, further contributing to the dysregulated immune response observed in MDS (43). Moreover, the overexpression of miR-34a has been implicated in reducing c-Fos levels, a factor that contributes to tumor necrosis factor-alpha (TNF-alpha) overproduction in response to inflammatory stimuli in MDS, ultimately leading to ineffective hematopoiesis (44). Furthermore, miR-34a has been implicated in inducing neutrophil apoptosis via the Cdc42-WASP-Arp2/3 pathway, contributing to the dysregulation of myeloid cell homeostasis in MDS (45). These multifaceted roles of miR-34a underscore its significance in MDS pathogenesis.

### MicroRNA-146b

The microRNA-146 family comprises two members, miR146a and miR146b, which are located on different chromosomes, but are nearly identical in sequence (46). Studies have revealed that these microRNAs are implicated in inflammatory diseases and tumorigenesis by downregulating key molecules essential for NF-kB activation through the inhibition of tumor necrosis factor receptor–associated factor 6 (TRAF6) and interleukin-1 receptor–associated kinase 1 (IRAK1) (47). Furthermore, microRNA-146b-5p has been identified as a significant player in erythropoiesis and megakaryopoiesis by targeting platelet-derived growth factor receptor alpha (48). Notably, in animal models, knockout mice lacking miR-146a and miR-146b have shown a predisposition to developing hematopoietic malignancies with age, with miR-146a knockout mice developing lymphomas and miR-146b knockout mice developing lymphomas and acute myeloid leukemia (49). In clinical studies, the expression of miR-146b-5p has emerged as a potential diagnostic marker for MDS, with elevated levels observed in MDS and aplastic anemia (AA) patients compared to healthy controls (15). Additionally, research by Choi et al. has demonstrated a correlation between the expression levels of miR-146b-5p in bone marrow mononuclear cells and the prognosis of MDS patients, with significantly higher expression detected in high risk MDS cases (16).

### MicroRNA-424

MicroRNA-424 is another potential molecule in the pathogenesis of MDS. It has a role as a regulator of monocyte and macrophage differentiation (50) and is also associated with aberrant endothelial cell proliferation by directly targeting VEGFR2 and influencing angiogenic processes (51). Notably, dysregulated expression of miR-424 has been observed in hematological diseases, such as its upregulation in AML with MN1 overexpression (52). Studies have revealed the potential diagnostic value of miR-424, with its expression levels serving as a distinguishing factor among different hematological conditions.

It is found out to be downregulated in severe AA compared to MDS (15). In another study, miR-424 levels were significantly reduced in cell lines derived from monoMAC patients with MDS (20). Research by Kunze et al. utilizing next-generation sequencing and SNP array analysis in formalin- fixed, paraffin-embedded (FFPE) bone marrow biopsies from MDS patients, unveiled reduced miR-424 expression (53). This was linked to a recurrent microdeletion in Xq26.3, resulting in the loss of PHF6 expression, a potential tumor suppressor gene. MiR-424 has also a potential for therapeutic response assessment in MDS patients treated with azacitidine and lenalidomide (21). While preliminary findings indicate promising avenues for miR-424, further investigations involving larger cohorts are warranted to validate its clinical utility in MDS management.

### MicroRNA-597

MicroRNA-597 is located on chromosome 8, and trisomy 8 is present in 5-7% of patients with MDS, making it the most common chromosomal gain abnormality in MDS (54). Moreover, miR-597 is found out to be dysregulated in various solid cancers such as hepatocellular carcinoma (55), non-small cell lung cancer (56), colorectal neoplasms (57), and breast cancer (58), implicating its role in tumorigenesis. Recent research conducted by Kang et al. unveiled elevated levels of miR-597 in MDS patients (59). They performed an *in vitro* study, which demonstrated that miR-597 mimics induce apoptosis by downregulating FOS like 2 (FOSL2), shedding light on the role of miR-597 in MDS pathogenesis.

### MicroRNA-218

MicroRNA-218 is also involved in carcinogenesis and it is known for targeting the tumor suppressor gene slit guidance ligand (SLIT2/3). It is correlated with clinical staging, prognosis and metastasis of solid tumors, which underscores its significance in disease development (60). A study by Zhang et al. demonstrated that hypermethylation of the SLIT2 promoter leads to the repression of mir-218 expression, which is associated with disease progression in MDS and it is predictive of poor prognoses in both MDS and AML (23). Furthermore, the overexpression of SLIT2-IT1/miR-218 demonstrated potent anti-leukemic effects by modulating cell proliferation, apoptosis, and colony formation both *in vitro* and *in vivo*, highlighting the therapeutic potential of microRNA-218 in combating hematologic malignancies.

### MicroRNAs in DLK1-DIO3 genomic region

The DLK1–DIO3 genomic region is located on chromosome 14 (14q32) and contains one of the largest miRNA clusters (54 miRNAs) in the human genome (61). These miRs exhibit dual roles as both oncogenic drivers and tumor suppressors, with frequent dysregulation observed across various cancer types. Their aberrant expression has been associated with disrupted apoptosis and suppression of proliferation (62). Furthermore, these miRNAs play a crucial role in regulating hematopoietic stem/progenitor cell (HSPC) differentiation, highlighting their involvement in hematopoietic development (63).

In another study by Merkerova et al., the expression levels of miRNAs within the DLK1-DIO3 region were investigated in patients with high-risk MDS and acute myeloid leukemia with myelodysplasia-related changes (AML-MRC) (22). Despite the small cohort size, intriguing findings emerged, with approximately half of the patients demonstrating increased expression of these miRNAs. Notably, following treatment with azacitidine a reduction in miRNA expression levels to near-normal was observed. Furthermore, the study revealed a correlation between the expression levels of these miRNAs in pretreatment samples and important clinical parameters, including bone marrow blast count, patient diagnoses, and outcomes such as overall survival (OS) and progression-free survival (PFS). High expression was related with AML-MRC and poor outcome and low expression was associated with MDS and favorable outcome. These findings underscore the potential utility of miRNAs within the DLK1-DIO3 region as biomarkers for disease prognosis and treatment response in high-risk MDS and AML-MRC patients.

## MicroRNAs and specific chromosome abnormalities

Chromosome abnormalities represent a hallmark feature in MDS, with specific MDS karyotypes potentially linked to distinct microRNA expression profiles. Understanding these associations is a point of several investigations (**Table 2**). For instance, Bousquet et al. demonstrated elevated expression levels of microRNA-125b in patients harboring the translocation (2;11)(p21;q23) compared to both healthy individuals and patients without this translocation (64). Notably, mature miR-125b originates from two distinct loci: miR-125b-1, derived from the long noncoding RNA (lncRNA)–MIR100HG (miR-100/let-7a-2/miR-125b-1) on chromosome 11, and miR-125b-2, derived from the miRNA cluster (miR-99a/let-7c/miR-125b-2) on chromosome 21 (68). The role of microRNA-125b is to block the myelomonocytic differentiation of cell lines *in vitro* (64), shedding light on its potential role in MDS pathogenesis.

**Table 2:** MicroRNAs associated with specific chromosome abnormalities.

MicroRNA	Sample source	Karyotype association	Expression	Function	Target	Implication	Reference
MiR-150	Bone marrow	MDS with del(5q)	Upregulated	Role in erythropoiesis, megakaryopoiesis	MYB, BC200	Potential therapeutic target	(34)
MiR-125b	Bone marrow	t(2;11)(p21;q23)	Upregulated	Block the myelomonocytic differentiation		Potential therapeutic target	(64)
MiR-194	Bone marrow	Trisomy 1	Upregulated	Apoptosis	p53	Prognostic marker	(4)
MiR-595	Bone marrow	Chromosome 7 abnormalities	Downregulated	Cellular proliferation, apoptosis, and defective ribosomal biogenesis	RPL27A	Potential therapeutic target	(65)
miR-449a, miR-300, miR-210, miR-874, miR-589, miR-451 miR-223, miR-128b, miR-342	Bone marrow	MDS with del(5q)	Downregulated			Diagnostic markers	(66, 67)
miR-196b, miR-451, miR-98, miR-34a, miR-10a miR-10b, miR-126, miR-99b miR-130a, miR-199a, miR-125a, miR-125b	Bone marrow	MDS with del(5q)	Upregulated			Diagnostic markers	(66, 67)

In another study, Choi et al. looked into the involvement of microRNAs encoded on chromosomes 8 and 1q in patients with MDS (4). They specifically examined the expression levels of nine microRNAs encoded on chromosome 8 and three on chromosome 1q. Their findings revealed increased expression of miR-194-5p in patients with trisomy 1, a chromosomal aberration frequently observed in MDS. Moreover, the expression levels of miR-194-5p correlated with OS, with lower levels of this microRNA significantly associated with decreased OS. The increased expression of miR-194 is known to inhibit cell growth and promote apoptosis by regulating p53 through the suppression of E3 ubiquitin–protein ligase Mdm2 (69). Interestingly, p53, in turn, has been reported to induce the expression of miR-194, suggesting a complex interplay between microRNAs and key regulatory pathways implicated in MDS pathogenesis.

Expanding on the exploration of microRNAs in MDS, Alkhatabi et al. investigated miR-595, located on chromosome 7q (65). MiR-595 is situated within one of the commonly deleted regions (CDR) identified in MDS with monosomy 7 (-7) or isolated loss of 7q (7q-). Their study revealed significant dysregulation of this microRNA in MDS patients with -7/-7q and those with complex karyotypes containing chromosome 7 anomalies, compared to patients with a normal karyotype. Furthermore, Alkhatabi et al. demonstrated that miR-595 regulates RPL27A, a ribosomal protein-coding gene. They found out that deficiency of RPL27A leads to both p53-dependent and independent effects, including attenuated cellular proliferation, apoptosis, and defective ribosomal biogenesis. These findings shed light on the intricate molecular mechanisms underlying MDS pathogenesis and highlight miR-595 as a potential therapeutic target in MDS patients with chromosome 7 abnormalities.

As part of their comprehensive investigation into microRNA dysregulation in MDS, Zuo et al. examined specific plasma microRNA expression patterns in patients with MDS harboring isolated del(7q)/-7 or del(20q), compared with other MDS cases (70). Their investigation unveiled eight microRNAs significantly differentially expressed in MDS with isolated del(7q)/-7, four of which are mapped on chromosome 7 - miR-96, miR-196b, miR-25, and miR-590. Additionally, thirteen microRNAs exhibited significant differences in MDS with isolated del(20q), with two of them located on the long arm of chromosome 20. Intriguingly, the levels of these microRNAs were notably higher in this subset of patients. These findings contribute to our understanding of the molecular landscape of MDS and highlight the potential utility of plasma microRNAs in cases with specific chromosomal abnormalities.

In MDS, the 5q syndrome represents a distinct subtype characterized by a deletion of part of the long arm of chromosome 5 (del(5q)). This subtype typically manifests with cytopenias, particularly anemia, and displays a favorable prognosis compared to other MDS subtypes. Furthermore, the presence of the del(5q) abnormality is associated with specific clinical and molecular features, including responsiveness to lenalidomide therapy, suggesting a unique pathogenic mechanism mediated by dysregulated microRNAs. A certain number of studies have demonstrated a specific miR signature associated with 5q- phenotype. Distinct microRNA profiles have been identified in 5q syndrome, shedding light on its pathogenesis and potential therapeutic targets. Studies have revealed upregulation of miR-199a and miR-125a, as demonstrated by two independent investigations (66, 71). Furthermore, Votavova et al. uncovered increased expression of miR-10a, miR-10b, miR-34a, miR-451, miR-223, alongside downregulation of miR-128b and miR-342 (66). Understanding the dynamic changes in microRNA expression profiles following treatment with lenalidomide is essential for unraveling the complex pathogenesis of 5q syndrome, offering insights into the molecular mechanisms underlying therapeutic responses. Merkerova et al. conducted a study investigating microRNA expression in response to lenalidomide treatment, revealing a notable increase in pro-apoptotic miR-34a and miR-34a* expression, which decreased over the course of lenalidomide exposure (67). MiR-34a is directly regulated by p53, acting as a pro-apoptotic transcriptional target that modulates the expression of specific genes targeted by p53 (72). Additionally, they observed that microRNAs initially unchanged, such as those located in the 14q32 locus and miR-145 and miR-146a, exhibited increased levels following treatment, suggesting dynamic regulatory shifts associated with therapeutic response (67). MiR-145 and miR-146a are notable for their location within the 5q chromosomal region. Starczynowski et al. research demonstrated that reduced levels of these miRNAs in murine models led to a phenotype resembling MDS (73), highlighting their potential significance in disease pathogenesis. A proposed model suggests that hemizygous deletion of the miR-145 and the protein-coding gene RPS14 may collaborate to increase megakaryocyte production, contributing to thrombocytosis in the 5q syndrome (74). Moreover, miR-145 targets FLI1, a megakaryocyte and erythroid regulatory transcription factor who plays a central role in megakaryopoiesis (74). However, contradictory findings arise from other studies that did not detect changes in the expression levels of these miRNAs in patients with 5q syndrome (66, 71). Interestingly, the observed increase in miR-145 and miR-146a levels following lenalidomide treatment (67) offers insight into Starczynowski’s findings, suggesting a dynamic regulatory response that may impact disease progression and therapeutic outcomes in MDS.

## Circulating microRNAs as less invasive biomarkers

The collective findings from these studies underscore the potential of miRNAs as promising diagnostic and prognostic biomarkers in MDS. The majority of these research primarily focuses on microRNAs isolated from bone marrow CD34+ cells, given their direct relevance to hematopoietic stem cell biology and disease pathogenesis. As we strive for less invasive diagnostic procedures, miRNAs could offer a non-invasive avenue, as they can be isolated from various materials including plasma, serum, and extracellular vesicles. Several research endeavors have explored the differential expression of miRNAs in plasma and serum samples from MDS patients, revealing distinct miRNA profiles that could serve as valuable indicators of disease status and prognosis. By elucidating the intricate molecular signatures associated with MDS progression, these studies contribute to the ongoing efforts to refine diagnostic and prognostic strategies for this heterogeneous disorder (**Table 3**).

**Table 3:** Differentially expressed circulating microRNAs.

Upregulated plasma microRNAs	Downregulated plasma microRNAs	Upregulated exosomal microRNAs	Downregulated exosomal microRNAs
miR-206, miR-34b, miR-503, miR-651, miR-655, miR-150	miR-16, miR-let-7a, miR-144, miR-25, miR-451, miR-493, miR-92a, miR-96, miR-27a, miR-199a,	miR-103a, miR-103b, miR-107, miR-221, miR-221, miR-130b, miR-378i, miR-3200, miR-423, miR-1193, miR-143	miR-426, miR-19b, miR-1180, miR-126, miR-382

In one retrospective analysis, researchers compared the levels of let-7a and miR-16 in plasma samples from patients with MDS and healthy controls (5). Significantly lower levels of both miRNAs were observed in MDS patients, and these levels were found to be predictive of both OS and PFS. Building upon these findings, Zuo et al. conducted a subsequent study involving 72 patients diagnosed with cytogenetically normal myelodysplastic syndrome and 12 healthy controls (70). The study aimed to profile plasma miRNA expression patterns in MDS and identify miRNAs that could potentially serve as prognostic biomarkers. Utilizing an array containing 800 miRNAs, 639 of which generated analyzable signals, the patients were stratified into groups based on their survival outcomes, with a cutoff of 30 months for OS. Through this analysis, seven highly differentially expressed microRNAs (let-7a, miR-144, miR-16, miR-25, miR-451, miR-651, and miR-655) were identified, all of which demonstrated correlations with patient survival.

Merkerova et al. conducted a comprehensive genome-wide miRNA profiling study, analyzing plasma samples from a discovery cohort consisting of 14 patients with MDS and 7 healthy controls, using a microarray containing 2,006 miRNAs (75). They identified 207 and 201 miRNAs in the MDS and control samples, respectively. Subsequently, the patients were stratified into lower and higher risk categories, and six hematopoiesis and/or oncology-related miRNAs (miR-16-5p, miR-27a-3p, miR-150-5p, miR-199-5p, miR-223-3p, and miR-451) were selected for validation in a cohort of 40 MDS patients and 20 healthy controls. In the validation cohort, plasma levels of miR-150-5p were elevated, while miR-16-5p, miR-27a-3p, miR-199a-5p, and miR-451a were decreased in MDS patients compared to healthy controls. Moreover, lower levels of miR-27a-3p, miR-199-5p, and miR-223-3p were identified in patients with higher-risk disease. Additionally, univariate analysis revealed a correlation between PFS and the levels of five miRNAs (miR-27a-3p, miR-150-5p, miR-199a-5p, miR-223-3p, and miR-451a), as well as between OS and miR-27a-3p and miR-223-3p.

MicroRNAs can be extracted from various sources including plasma, extracellular vesicles (EVs), and bone marrow, with specific small RNAs selectively packaged within EVs (76). Hrustincova et al. conducted a study comparing the levels of miRNAs in total plasma with those in EVs, revealing substantial variability between the two types of material (77). Interestingly, the content of miRNAs in EVs was found to be more homogenous than that of total plasma. Furthermore, the RNA profiles of total plasma and paired EVs exhibited differences among MDS patients but remained similar in healthy controls. Specifically, numerous hematopoiesis-related miRNAs, such as miR-103a-3p, miR-103b, miR-107, miR-221-3p, miR-221-5p, and miR-130b-5p, were elevated in both plasma and EVs of MDS patients compared to healthy controls. Intriguingly, several dysregulated miRNAs, including miR-127- 3p, miR-154-5p, miR 323-3p, miR-383-3p, miR-409-5p, and miR-485-3p, clustered on chromosomal region 14q32, were significantly upregulated in early MDS stages. This specific increase in miRNA expression in early MDS may be linked to excessive apoptosis, while advanced MDS is characterized by the inhibition of apoptosis and a proproliferative phenotype (78). These findings shed light on the dynamic role of miRNAs in different stages of MDS pathogenesis. Another research conducted by Giudice et al. investigated exosomal microRNAs in patients with AA and MDS (79). Their analysis revealed 25 exosomal miRs uniquely or commonly present in AA and/or MDS patients. Notably, 14 exosomal miRNAs were exclusively present in MDS patients, while 7 miRNAs were common to both SAA and MDS. This study underscores the potential of exosomal miRNAs as biomarkers for differentiating between these hematologic conditions and understanding their underlying pathophysiology.

When comparing cellular, plasma, and exosomal miRNAs, each type has distinct advantages and challenges. Cellular miRNAs can be isolated from CD34+ bone marrow cells, making them highly specific for the pathology of MDS. This specificity is invaluable for detailed research, and indeed, a substantial body of research focuses on these miRNAs. However, isolating cellular miRNAs is a more invasive procedure, which for example might complicate treatment response assessment due to the need for repeated bone marrow aspirations. On the other hand, plasma and exosomal miRNAs can be conveniently obtained from blood samples, offering a less invasive alternative. Exosomal miRNAs, in particular, are considered more reliable because they are tissue-specific, protected from degradation, and specifically loaded into vesicles from proliferating or apoptotic cells (80, 81). Nevertheless, there are technical challenges associated with exosome preparation and RNA extraction (82), making them harder to obtain. Despite their potential, the lack of clear guidelines for data normalization in exosomal miRNA research remains a significant limitation (83). In contrast, data normalization for circulating plasma miRNAs is well-established. It typically employs small nucleolar RNAs (snoRNAs) and other robust methods like GeNormPlus, NormFinder, and the global mean of miRNA expression (79). This standardization makes plasma miRNA data more accessible and reliable for clinical and research applications, despite the broader and potentially less specific nature of plasma-derived miRNAs.

## MicroRNAs as markers for response to therapy

Hypomethylating agents are used as first-line therapy in patients with high-risk MDS, with azacitidine being preferred, resulting in better overall survival. Azacitidine prolongs patient survival, improves quality of life and delays time to AML progression. However it takes several months to determine the effect of the therapy and the response to the treatment occurs in approximately 40-50% of patients (84, 85). This necessitates the need for biomarkers to determine the sensitivity of patients to this type of therapy.

Certain miRs are associated with different response to azacitidine treatment. For example the plasma levels of miR-4474-3p and miR-762 are increased and the levels of miR-125b-5p, miR4324, miR-3156-3p are decreased in relation to later response (77).

In a study conducted by Mongiorgi et al., the expression of miR-192-5p in MDS patients treated with azacitidine and lenalidomide was investigated. Their findings revealed a consistent increase in miR-192-5p levels in MDS patients compared to healthy controls (86). MiR-192-5p, belonging to the miR-192/215 family, is recognized as a conserved tumor and leukemia-related miRNA (87). It exerts a tumor-suppressive role by targeting key genes involved in oncogenic pathways, including BCL2, TP53, and TGF-beta signaling (88–90) while inhibiting CCNT2 in leukemia (91), thereby suppressing cell proliferation and inducing G0/G1 cell cycle arrest in AML cells (92). Interestingly, differences in miR-192-5p expression were noted between responders and patients who lost response early and therapy non-responders (86). Furthermore, after four cycles of therapy, a statistically significant upregulation of miR-192-5p was observed, suggesting its association with treatment response. Additionally, Mongiorgi et al. demonstrated that miR-192 targets the BCL2 promoter, suggesting a potential mechanism underlying the suppressive role of miR-192-5p, as evidenced by the low BCL2 gene expression in MDS responder patients, possibly correlating with inhibition of proliferation in this subgroup. These findings underscore the intricate regulatory role of miR-192-5p in MDS pathogenesis and treatment response, offering valuable insights into its therapeutic potential and prognostic significance in this disease context.

MicroRNAs have also a role in chemoresistance, as evidenced by several studies in the field. For instance, Solly et al. investigated microRNAs associated with azacitidine resistance and identified seven microRNAs that were downregulated in azacitidine-resistant SKM1 cells, with five of these targeting DNMT1, a key enzyme inhibited by azacitidine. Among these microRNAs, miR-126-3p showed the most significant prognostic impact, with low levels correlating with reduced response rates, increased relapse rates, and shorter progression-free survival and overall survival (93). Notably, miR-126 interacts with DNMT1 to suppress its translation without affecting its transcription (94). In another study, Lei et al. explored decitabine (DAC) resistance and revealed that miR-4755-5p is overexpressed in extracellular vesicles from a DAC-resistant cell line (KG1a-DAC), promoting resistance by targeting the CDKN2B gene (95). Additionally, Li et al. demonstrated that both high and low levels of miR92a-Exos can induce chemoresistance to Ara-C in recipient cells, with low miR92a-Exos levels leading to a less pronounced resistance in SKM1 cells. Their findings highlighted the role of miR-92 in targeting PTEN and activating the Wnt/beta- catenin pathway to induce Ara-C resistance (96). These studies collectively underscore the intricate involvement of microRNAs in mediating chemoresistance mechanisms in various contexts (**Table 4**).

**Table 4:** MicroRNAs with implications in therapy.

MicroRNA	Sample source	Karyotype association	Expression	Function	Target	Implication	Reference
MiR-22	Bone marrow/Plasma		Upregulated	Role in HSC renewal	TET2	Prognosis/Therapeutic target	(14)
MiR-34a	Peripheral blood		Upregulated	Regulator of proliferation	DOCK8, FGD4, Rac1, c-Fos, Cdc42-WASP-Arp2/3 pathway	Potential therapeutic target	(43–45)
MiR-150	Bone marrow	MDS with del(5q)	Upregulated	Role in erythropoiesis, megakaryopoiesis	MYB, BC200	Potential therapeutic target	(34)
MiR-765	Bone marrow		Upregulated	Apoptosis	PLP2	Potential therapeutic target	(40)
MiRs in DLK1-DIO3 genomic region	Bone marrow		Upregulated	Apoptosis, regulation of HSPC differentiation	MEG3-DMR	Prognostic markers, Therapy response markers	(22)
MiR-125b	Bone marrow	t(2;11)(p21;q23)	Upregulated	Block the myelomonocytic differentiation		Potential therapeutic target	(64)
MiR-218			Downregulated	Role in tumorigenesis	SLIT2/3	Prognostic marker, Potential therapeutic target	(23)
MiR-595	Bone marrow	Chromosome 7 abnormalities	Downregulated	Cellular proliferation, apoptosis, and defective ribosomal biogenesis	RPL27A	Potential therapeutic target	(65)
MiR-597			Upregulated	Apoptosis	FOSL2	Potential therapeutic target	(59)
MiR-192	Bone marrow/plasma		Upregulated	Tumor suppressor	BCL2, TP53, and TGF-beta	Therapy response	(86)
MiR-126	Bone marrow		Downregulated		DNMT1	Azacitidine resistance	(93)
MiR-4755			Upregulated		CDKN2B	Decitabine resistance	(95)
MiR-92a	Exosomes		Upregulated		PTEN	Ara-C resistance	(96)
MiR-21	Bone marrow		Upregulated		SMAD7, TGF-b	Therapeutic target	(97)
MiR-146a					TRAF6, IRAK1	Therapeutic target	(98)

## MicroRNAs as therapeutics

MicroRNAs can not only be used as diagnostic and prognostic biomarkers but also as therapeutic targets. These small, non-coding RNA molecules can act as tumor suppressors or oncogenes (oncomiRs) and have the ability to target multiple mRNAs that may be dysregulated in various diseases, making them promising candidates for therapeutic targets (**Table 4**). MicroRNA-based therapeutics can be broadly classified into two categories: microRNA mimics and microRNA inhibitors or antimiRs (99, 100). MicroRNA mimics are designed to replenish the lost miRNA expression in disease, thereby restoring normal cellular function. On the other hand, microRNA inhibitors or antimiRs are synthetic molecules with sequences complementary to the miRNA to be inhibited. By binding strongly to their target miRNAs, antimiRs effectively block the miRNAs function, thereby preventing its pathological effects.

The development of miRNAs as therapeutic agents presents several challenges, which must be addressed to ensure their efficacy and safety. One of the primary difficulties is the stability of miRNAs inside the body because they are susceptible to degradation by RNases present in the serum or within cellular endocytic compartments. One strategy to address this challenge is to modify the structure of oligonucleotides by altering the nucleotides or RNA backbone. Another strategy focuses on developing delivery vehicles, such as lipid nanoparticles, to encapsulate RNAs, offering protection and endosomal escape (100). Another concern is determining the proper administration routes to deliver miRNAs effectively to the target sites (101). Additionally, identifying the most suitable miRNA candidate for each disease remains a significant challenge. Addressing this issue requires a systematic analysis of existing data on various miRNA profiles across different diseases, and a thorough understanding of the miRNA-target networks involved in disease pathogenesis. By overcoming these hurdles, the potential of miRNAs as powerful therapeutic tools can be realized.

There are currently a few Phase I and II clinical trials testing these innovative therapeutic approaches involving miRNAs. One notable trial involves miravirsen, a 15-nucleotide antisense RNA oligo that targets miR-122-5p, designed for the treatment of Hepatitis C Virus (HCV) (102). Additionally, MRX34, a miR-34 mimic developed by Mirna Therapeutics, was encapsulated in a lipid carrier (103) and entered a multicenter Phase I trial in 2013. This trial included patients with advenced solid tumours (104). Unfortunately, the trial was terminated due to immune-related adverse events, including patient deaths (105). The exact cause of these immune reactions remains unclear, highlighting the need for redesigned pre-clinical trials with a focused study on immune-related toxicities. Furthermore, another Phase I clinical trial was initiated for the LNA-based antimiR-155 (MRG-106; miRagen Therapeutics) in patients with cutaneous T cell lymphoma, mycosis fungoides subtype with promising preliminary results (106).

**Figure 2:**
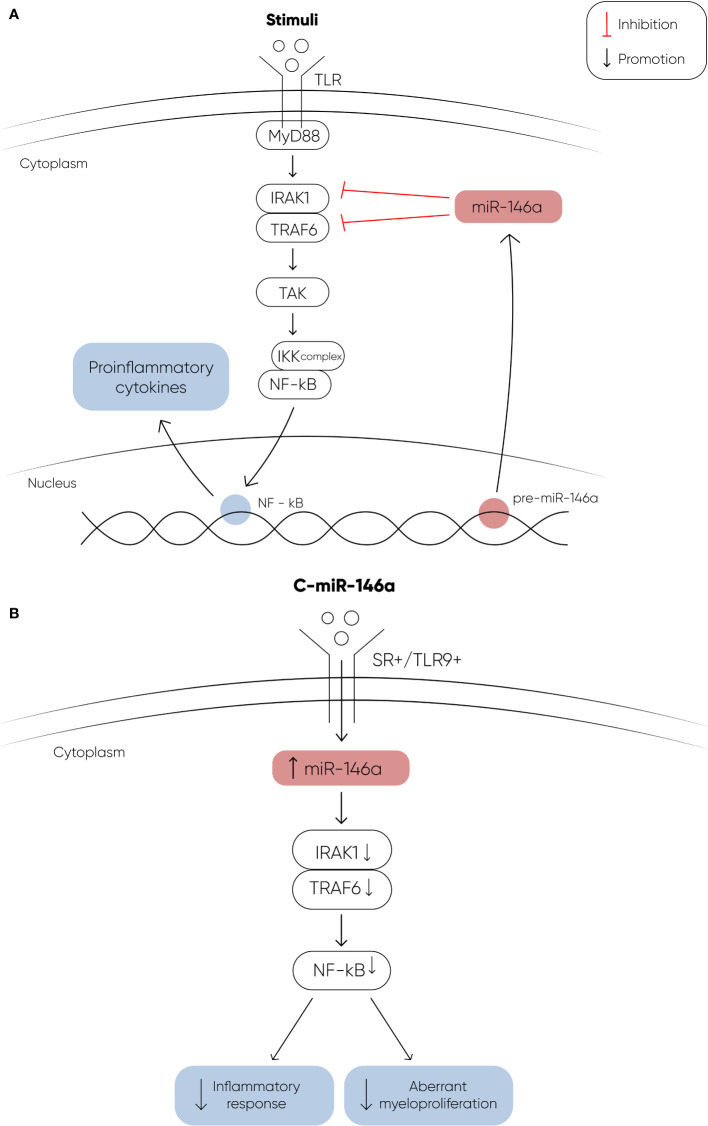
**(A)** The figure depicts the signaling cascade initiated by Toll-like receptors (TLRs) upon ligand binding, and the regulatory role of miR-146a in this pathway. Upon binding to their ligands, TLRs undergo a conformational change that recruits the adapter protein MyD88, leading to the activation of IRAK1. Activated IRAK1 then binds to TRAF6, which in turn activates TAK1. TAK1 phosphorylates the IKK complex, resulting in the activation of the transcription factor NF-κB. Activated NF-κB translocates into the nucleus to induce the expression of proinflammatory genes. MiR-146a targets and downregulates IRAK1 and TRAF6, thereby modulating this signaling pathway. In MDS, decreased levels of miR-146a contribute to the activation of NF-κB through IRAK1 and TRAF6, promoting the development of MDS and its progression to AML. **(B)** The therapeutic potential of a chemically modified miRNA-146a mimic oligonucleotide (C-miR146a) conjugated to a scavenger receptor/Toll-like receptor 9 agonist. This conjugation significantly increases the levels of miR-146a, effectively restoring its function. The restoration of miR-146a levels results in near-complete and durable inhibition of its targets, IRAK1 and TRAF6. This leads to the complete elimination of exacerbated NF-κB activity, thereby preventing exaggerated inflammatory responses and aberrant myeloproliferation (107, 108).

The potential utilization of miRNA therapeutics in myeloid malignancies is gaining significant interest, particularly with promising research findings. In a study conducted by Bhagat et al, researchers discovered elevated levels of miR-21, a microRNA that binds to SMAD7, a negative regulator of transforming growth factor–beta (TGF-b) receptor-I kinase, leading to reduced expression in hematopoietic cells (97). To explore the implications of this finding, they treated transgenic mice expressing a fusion gene (Alb/TGF) with chemically modified inhibitors of miR-21. Remarkably, the treated mice were with significantly increased hematocrit levels and showed enhanced capacity to form erythroid colonies from marrow-derived cells. The researchers further tested miR-21 inhibitors in primary MDS bone marrow samples, observing a notable increase in erythroid (BFU-E) colony numbers. These findings highlight the potential of miR-21 as a therapeutic target in hematologic disorders, suggesting a promising avenue for future research and clinical applications. Another recent study explored the therapeutic effects of a chemically modified miRNA-146a mimic oligonucleotide conjugated to a scavenger receptor/Toll-like receptor 9 agonist (C-miR146a) in miR-146a knockout mice (98). Reduced expression of miR-146a is known to contribute to the development of del(5q) MDS (73) and its progression to AML through IRAK1- and TRAF6-dependent activation of NF-kB (107, 108). In this study, intravenous injection of C-miR146a successfully restored miR-146a-5p levels in target myeloid cells, achieving complete elimination of exacerbated NF-kB activity in miR-146a knockout mice. This restoration prevented exaggerated inflammatory responses and aberrant myeloproliferation, leading to near-complete and durable inhibition of classic miR-146a targets, IRAK1 and TRAF6 (**Figure 2**). Furthermore, C-miR146a demonstrated cytotoxic effects on human MDSL, HL-60, and MV4-11 leukemia cells *in vitro*. Repeated intravenous administration of C-miR146a inhibited the expression of NF-kB target genes, thereby thwarting the progression of disseminated HL-60 leukemia. These findings underscore the therapeutic potential of miRNA mimics in treating myeloid malignancies, highlighting the ability to correct specific molecular dysfunctions and inhibit key pathological pathways.

## Conclusions

In summary, the dysregulation of microRNAs in MDS patients underscores their potential as crucial players in disease pathogenesis. With distinct miRNA profiles associated with various types of MDS, these small regulatory molecules offer promise as less invasive diagnostic and prognostic biomarkers. Moreover, their potential as indicators for response to therapy suggests a future role in treatment decision-making and monitoring. However, despite significant progress, there remain many unanswered questions, highlighting the need for further research in larger cohorts of patients. Continued exploration of miRNA dysregulation in MDS holds the key to unlocking novel insights into disease mechanisms and refining personalized approaches to patient care.

## Author contributions

IM: Conceptualization, Supervision, Writing – original draft, Writing – review & editing. SA: Conceptualization, Methodology, Writing – original draft.
