# Supplementary material for: MicroRNA dysregulation in myelodysplastic syndromes: implications for diagnosis, prognosis, and therapeutic response
Source: Front Oncol. 2024 Aug 2;14:1410656. doi: 10.3389/fonc.2024.1410656 (PMC11327013; doi:10.3389/fonc.2024.1410656)
Supplement: Supplementary file 1 [file DataSheet_1.pdf]

Supplementary Table 1: Summary of microRNAs with implication in MDS.

MicroRNA	Sample source	Karyotype association	Expression	Function	Target	Implication	Reference
MiR-16	Bone marrow		Upregulated	Tumor suppressor	VEGF	Prognosis	[13]
MiR-22	Bone marrow/ Plasma		Upregulated	Role in HSC renewal	TET2	Prognosis/ Therapeutic target	[19]
MiR-34a	Peripheral blood		Upregulated	Regulator of proliferation	DOCK8, FGD4, Rac1, c-Fos, Cdc42-WASP-Arp2/3 pathway	Potential therapeutic target	[38], [39], [40]
MiR-146b	Bone marrow		Upregulated	Role in tumorigenesis, erythropoiesis, megakaryopoiesis	TRAF6, IRAK1, PDGFRA	Diagnostic and prognostic marker	[45], [46]
MiR-150	Bone marrow	MDS with del(5q)	Upregulated	Role in erythropoiesis, megakaryopoiesis	MYB, BC200	Potential therapeutic target	[25]
MiR-181	Bone marrow		Upregulated	Regulator of granulocytic and macrophage differentiation	PRKCD, CTDSPL, CAMKK1	Prognostic marker	[7], [28], [29]
MiR-320	Bone marrow		Upregulated			Diagnostic and prognostic marker	[35],[36]
MiR-424	Bone marrow		Downregulated	Regulator of monocyte	VEGFR2	Diagnostic marker,	[45], [50], [52]

				and macrophage differentiation		therapy response marker	
MiR-765	Bone marrow		Upregulated	Apoptosis	PLP2	Potential therapeutic target	[33]
MiRs in DLK1-DIO3 genomic region	Bone marrow		Upregulated	Apoptosis, regulation of HSPC differentiation	MEG3-DMR	Prognostic markers, Therapy response markers	[64]
MiR-125b	Bone marrow	t(2;11)(p21; q23)	Upregulated	Block the myelomonocytic differentiation		Potential therapeutic target	[65]
MiR-194	Bone marrow	Trisomy 1	Upregulated	Apoptosis	p53	Prognostic marker	[36]
MiR-218			Downregulated	Role in tumorigenesis	SLIT2/3	Prognostic marker, Potential therapeutic target	[60]
MiR-595	Bone marrow	Chromosome 7 abnormalities	Downregulated	Cellular proliferation, apoptosis, and defective ribosomal biogenesis	RPL27A	Potential therapeutic target	[68]
MiR-597			Upregulated	Apoptosis	FOSL2	Potential therapeutic target	[58]
MiR-192	Bone marrow /plasma		Upregulated	Tumor suppressor	BCL2, TP53, and TGF-beta	Therapy response	[88]
MiR-126	Bone marrow		Downregulated		DNMT1	Azacitidine resistance	[95]

MiR-4755			Upregulated		CDKN2B	Decitabine resistance	[97]
MiR-92a	Exosomes		Upregulated		PTEN	Ara-C resistance	[98]
MiR-21	Bone marrow		Upregulated		SMAD7, TGF-b	Therapeutic target	[107]
MiR-146a					TRAF6, IRAK1	Therapeutic target	[108]
miR-449a, miR-300, miR-210, miR-874, miR-589, miR-451 miR-223, miR-128b, miR-342	Bone marrow	MDS with del(5q)	Downregulated			Diagnostic markers	[70], [72]
miR-196b, miR-451, miR-98, miR-34a, miR-10a miR-10b, miR-126, miR-99b miR-130a, miR-199a, miR-125a, miR-125b	Bone marrow	MDS with del(5q)	Upregulated			Diagnostic markers	[70], [72]
miR-206, miR-34b, miR-503, miR-651, miR-655, miR-150	Plasma		Upregulated			Diagnostic and prognostic markers	[69], [77]
miR-16, miR-let-7a, miR-144, miR-25, miR-451, miR-493, miR-92a, miR-96,	Plasma		Downregulated			Diagnostic and prognostic markers	[69], [76], [77]

miR-27a, miR-199a,							
miR-103a, miR-103b, miR-107, miR-221, miR-221, miR-130b, miR-378i, miR-3200, miR-423, miR-1193, miR-143	Exosomes		Upregulated			Diagnostic and prognostic markers	[79], [81]
miR-426, miR-19b, miR-1180, miR-126, miR-382	Exosomes		Downregulate d			Diagnostic and prognostic markers	[81]
